# RACE, BIOLOGICAL AGE, AND COGNITION: THE SYSTEMATIC ASSESSMENT OF GERIATRIC ELEMENTS IN ATRIAL FIBRILLATION STUDY

**DOI:** 10.1093/geroni/igz038.1175

**Published:** 2019-11-08

**Authors:** Sarah N Forrester, David D McManus, Jane S Saczynski, Catarina I Kiefe

**Affiliations:** 1 University of Massachusetts Medical School, Worcester, Massachusetts, United States; 2 Northeastern University, Boston, Massachusetts, United States

## Abstract

Atrial Fibrillation (AF) is associated with dementia and cognitive decline. AF is less prevalent among Blacks than Whites, although AF-related complications are more common in Blacks. In the general population, all-cause cognitive decline and dementia are more prevalent among Blacks than Whites. Thus, studying diverse populations with AF may advance our understanding of racial disparities in cognitive functioning. We created a measure of multisystem dysregulation (weathering), which includes but is more encompassing than aging, and examined its association with racial differences in cognition using data from the SAGE-AF study, a prospective cohort of >65-year olds with AF, at high stroke risk, and eligible for anticoagulation. Biological (as opposed to chronological) age among 974 participants was calculated using the Klemera and Doubal method using biomarkers representing physiological functioning, metabolism, and blood pressure. We defined weathering as the difference between biological and chronological age (weathering >0 indicates that biological age is higher than chronological age). We measured the association between weathering and the Montreal Cognitive Assessment (MoCA) score. Mean weathering (SD) was -0.7 (11.5) and 4.3 (12.6) for whites and non-whites, respectively. There was an interaction between race/ethnicity and weathering on cognition (P=0.004). In stratified analyses, higher weathering was associated with a lower MoCA score among both Whites and non-Whites but more so among non-whites (B = -0.09, 95% CI: -0.17, -0.02) for Whites (B = -0.03, 95% CI: -0.06, -0.01) for non-whites. Aging-related multisystem dysregulation is more strongly associated with worse cognition in non-whites than in whites.

